# Characterization of Physicochemical, Biological, and Chemical Changes Associated with Coconut Milk Fermentation and Correlation Revealed by ^1^H NMR-Based Metabolomics

**DOI:** 10.3390/foods12101971

**Published:** 2023-05-12

**Authors:** Wasim S. M. Qadi, Ahmed Mediani, Khaled Benchoula, Eng Hwa Wong, Norazlan Mohmad Misnan, Norrakiah Abdullah Sani

**Affiliations:** 1 Department of Food Science, Faculty of Science and Technology, Universiti Kebangsaan Malaysia, Bangi 43650, Malaysia; 2 Institute of Systems Biology (INBIOSIS), Universiti Kebangsaan Malaysia, UKM Bangi, Bangi 43600, Malaysia; 3 School of Medicine, Faculty of Health and Medical Sciences, Taylor’s University, 1, Jalan Taylors, Subang Jaya 47500, Malaysia; 4 Herbal Medicine Research Centre, Institute for Medical Research, National Institutes of Health, Shah Alam 40170, Malaysia

**Keywords:** fermentation, coconut milk, antioxidant activity, antibacterial activity, storage, metabolomics, ^1^H NMR

## Abstract

Fermentation of milk enhances its nutritional and biological activity through the improvement of the bioavailability of nutrients and the production of bioactive compounds. Coconut milk was fermented with *Lactiplantibacillus plantarum* ngue16. The aim of this study was to evaluate the effect of fermentation and cold storage for 28 days on physicochemical characteristics, shelf life, and antioxidant and antibacterial activities of coconut milk as well as its proximate and chemical compositions. The pH of fermented milk decreased from 4.26 to 3.92 on the 28^th^ day during cold storage. The viable cell count of lactic acid bacteria (LAB) in fermented coconut milk was significantly increased during fermentation and cold storage period (1 to 14 days), reaching 6.4 × 10^8^ CFU/mL, and then decreased significantly after 14 days to 1.6 × 10^8^ CFU/mL at 28 days. Yeast and molds in fermented coconut milk were only detected on the 21^st^ and 28^th^ days of cold storage, which ranged from 1.7 × 10^2^ to 1.2 × 10^4^ CFU/mL, respectively. However, the growth of coliforms and *E. coli* was observed on the 14^th^ until the 28^th^ day of cold storage. The fermented coconut milk demonstrated strong antibacterial activity against *Staphylococcus aureus*, *Bacillus subtilis*, *Escherichia coli*, *Cronobacter sakazakii*, *Bacillus cereus*, and *Salmonella typhimurium* compared to fresh coconut milk. Fermented coconut milk had the greatest 1,1-diphenyl-2-picrylhydrazyl (DPPH) and ferric reducing antioxidant power (FRAP) values, with 67.1% and 61.961 mmol/g at day 14 of cold storage, respectively. Forty metabolites were detected in fermented and pasteurized coconut milk by proton nuclear magnetic resonance (^1^H NMR) metabolomics. The principal component analysis (PCA) showed clear difference between the fermented and pasteurized coconut milk as well as the studied cold storage days. The metabolites responsible for this variation were ethanol, valine, GABA, arginine, lactic acid, acetoin, alanine, phenylalanine, acetic acid, methionine, acetone, pyruvate, succinic acid, malic acid, tryptophan, uridine, uracil, and cytosin, which were higher in fermented coconut milk. However, sugars and other identified compounds were higher in fresh coconut milk. The findings of this study show that fermentation of coconut milk with *L. plantarum* ngue16 had high potential benefits to extending its shelf life and improved biological activities as well as other beneficial nutrients.

## 1. Introduction

Fermentation is an ancient technology for extending food’s shelf life and improving its nutritional and organoleptic properties. During fermentation, several biochemical changes may occur, resulting in a changed ratio of nutritive and antinutritive components, which influences the product’s bioactivity and digestibility [1]. This bioprocess has recently been used in the food, chemical, and pharmaceutical sectors to produce and extract bioactive chemicals. Furthermore, bioactive chemicals produced during the fermentation process (vitamins, antioxidant compounds, peptides, and phenolic compounds) boost the antioxidant and antibacterial activity of foodstuffs [1]. Milk is a nutrient-rich beverage that humans consume to achieve and satisfy their nutritional needs. It is sourced from cows, goats, and plant sources such as soy, rice, almonds, and coconut [2]. Nowadays, the nutritional profile of plant-based drinks, including plant-based milk, has become relevant to consumers for various reasons. These include allergies to cow’s milk, lactose intolerance, and concerns about the presence of growth hormones or antibiotic residues in cow’s milk [2]. The fermentation of nondairy milk products was mostly performed using lactic acid bacteria (LAB) to generate probiotic fermented milks and has become more popular. The manufacturing of high-quality fermented milk containing probiotic bacteria is a formidable obstacle due to technological issues, legislative aspects, ability of probiotics to multiply in food, safety of product, unknown health benefits, as well as consumer demands.

The LAB is used to produce fermented milk products such as yoghurt and cheese, fermented vegetables, including sauerkraut, cucumber pickles, and olives as well as fermented fish and meat [3]. The significance of LAB in food fermentation and preservation against pathogenic microorganisms is crucial. This is owing to the capacity of LAB to make organic acids, such as lactic acid and acetic acid, as well as to enhance the texture and taste of many milk and fermented food items and preserve their nutritional content. Fermented milk products benefit human health via a variety of mechanisms. For instance, *Lb. helveticus* produces peptide from casein milk protein and has antihypertensive, immunological modulator, and anticancer properties [4]. Several studies have investigated the development and viability of probiotics in fermented plant-based milk products throughout the fermentation and storage processes. Due to its high nutritional content, which promotes the development of probiotic bacteria, coconut milk is suggested as a substitute for dairy milk in probiotic formulations [4]. Coconut milk intake is seldom connected to allergic responses, aids digestion, nourishes the skin and hair, and is associated with anticarcinogenic, antimicrobial, antioxidative, and antiviral effects [5]. Coconut oil is high in lauric acid and phenolic compounds produced by the fermentation of coconut milk [6]. Coconut milk may also be made into other dairy products, such as yoghurt, which provides substantial advantages to consumers [7]. Fermented coconut milk may be stored for a long period because of the presence of natural additives accumulated during fermentation, including organic acids, peptides, and phenolic compounds. Additionally, fermented coconut milk provides various health advantages over fresh coconut milk and involves many biological and chemical changes.

The study of all the metabolites produced by an organism when it is exposed to various conditions is known as metabolomics [8]. For the high throughput study of targeted and untargeted metabolites, many cutting-edge technologies have been employed in metabolomics. The simultaneous identification of the several classes of secondary metabolites as well as the numerous primary metabolites makes nuclear magnetic resonance (NMR) an adequate and appropriate method for these investigations, integrated with multivariate data processing. Additionally, this technique requires straightforward sample preparation and is quick and repeatable. The NMR technique has been used for metabolic profiling and characterization of various fermented samples. There is, however, no information available regarding the bioactivity and metabolic differentiation of fermented and nonfermented coconut milk at varied cold storage times utilizing this advance technology. The purpose of this study is to address the knowledge gap on the chemical and biochemical properties of fermented coconut milk. To this aim, we investigated the changes of pH, acidity, and the most significant microbial groups during cold storage of fermented and pasteurized coconut milk. We characterized the chemical composition of both types of milk as well as antioxidant and antibacterial activities. Finally, we used an NMR-based metabolomics approach to explore the composition of the methanol extract and possible correlation with the biological activity of fermented coconut milk.

## 2. Materials and Methods

### 2.1. Sample

*Lactiplantibacillus plantarum* ngue16 and pathogenic *S. aureus* ATCC^®^25923™, *B. subtilis* ATCC^®^6633™, *E. coli* O157:H7 IMR E91, *C. sakazakii* ATCC^®^25944™, Bacillus cereus ATCC^®^33019, and *Salmonella Typhimurium* ATCC^®^14028™ were obtained from the Food Science laboratory, Faculty of Science and Technology, *Universiti Kebangsaan Malaysia* (UKM). Coconut milk was obtained from the market in Bandar Baru Bangi. The *Lactiplantibacillus plantarum* ngue16 strains were proliferated 3 times (1% inoculum) in MRogosa Sharpe (MRS) broth (Merck^®^, Darmstadt, Germany) and incubated anaerobically at 37 °C for 24 h, whereas pathogenic strains were subcultured three times (1% inoculum) and incubated aerobically for 24 h in Brain Heart Infusion (BHI) broth (Himedia^®^, Mumbai, India) at 37 °C. The stock cultures were frozen at −80 °C in the presence of 40% glycerol for further use.

### 2.2. Preparation of Fermented Coconut Milk

Fermentation of coconut milk was carried out following the previous method with some modifications [9]. The fresh coconut milk was filtered twice with cheese cloth and subjected to homogenization at 40/4 MPa followed by pasteurisation at 90 °C for 3 min prior to chilling at 4 °C before fermentation. *Lactiplantibacillus plantarum* ngue16 were activated (1%) in MRS broth incubated at 37 °C for 24 h, centrifuged at 5600 rpm at room temperature, and the cells were washed twice with saline solution 0.85% (*w*/*v*) and then suspended in the same solution. The fermentation of coconut milk was performed by inoculation of 2% (*v*/*v*) activated probiotic culture at initial inoculum density of approximately 10^6^ CFU mL^−1^ into coconut milk. The control was coconut milk prepared using the same conditions without the addition of the starter culture. The samples were incubated at 33 °C for 15 h, and after fermentation, the samples were stored at 4 °C for one month for further analysis.

### 2.3. pH and Total Titratable Acidity (TTA) during Storage

The pH of fermented milk samples was measured with a pH meter (Mettler-Toledo^®^, Schwerzenbach, Switzerland). The titratable acidity was determined using the AOAC method [10]. A total of 25 mL of the fermented coconut milk and pasteurized coconut milk was measured into conical flasks, and a few drops of 1% phenolphthalein indicator (2 mL) were added to each sample and titrated with 0.1 M NaOH to the first permanent pink color. The acidity was reported as the percentage of lactic acid by weight.

Titratable Acidity (%) = Quantity of NaOH (mL) × 0.009 × 100/Quantity of sample.

### 2.4. Microbial Analysis

The enumeration for LB was performed to determine the viable cells count in the fermented coconut milk after prolonged cold storage following the previous method [11]. Fermented coconut milk and the pasteurized coconut milk (25 mL) were subjected to serial dilution with 225 mL of maximum recovery diluent (MRD). Serial dilution was persisted until 10^6^ dilution was obtained. The inoculation of the sample (0.1 mL) was performed on MRS agar, and the plates were anaerobically incubated at 37 °C for 48 h using anaerobic jar and anaerogen sachets (Oxoid). The MRS plates showing 30–300 colonies were used to determine the viable cells that were expressed as Log CFU/mL using the formula:

N (CFU/mL) = C/v (n1 + 0.1 n2)d.

C: Sum of colonies on the plate.

v: Volume applied to each plate (0.1 mL).

n1: Number of plates counted at the first dilution.

n2: Number of plates counted at the second dilution.

d: Dilution from which the first count was obtained.

For mold and yeast count, the above procedure was repeated by the spread plate method using dichloran rose-bengal chloramphenicol (DRBC) agar, and incubation was performed at 25 °C for 72 h for coliform and *E. coli* using coliform agar [11]. The fermented coconut milk and pasteurized milk were counted in triplicate every week for four weeks.

### 2.5. Proximate Analysis of Fermented and Fresh Coconut Milk

Chemical composition of fermented coconut milk and pasteurized coconut milk were determined. Oven drying was utilized to determine the moisture content. The protein content was determined using the Kjeldahl method and a 6.25-fold conversion factor. After acid hydrolysis, the fat content was determined by solvent extraction. The amount of ash was measured using a muffle furnace. Carbohydrates were computed using the difference method.

### 2.6. Extraction of Bioactive Compounds from Fermented and Nonfermented Coconut Milks

A volume of 20 mL from fermented and pasteurized coconut milk was combined with methanol (100 mL), and the mixture was shaken at room temperature for 3 h using an orbital shaker at 90 rpm. The mixture was filtered through Whatman paper no. 4, and the liquid extract was evaporated under vacuum at 50 °C using a rotary evaporator (Rotavapor R−114, Buchi, Switzerland) before it was freeze dried to remove any moisture. The dried extracts were kept in an amber bottle at 4 °C before use. All extractions were performed in 5 replicates. The concentrated extract was volumetrically adjusted with methanol to 10 mL in a volumetric flask for further bioassay analyses.

### 2.7. Antibacterial Activity of Fermented Coconut Milk

#### 2.7.1. Determination of Antibacterial Activity by Microtiter Plate Assay

The assay was performed using three gram-positive bacterial strains (*S. aureus* ATCC^®^25923™, *B. subtilis* ATCC^®^6633™, and *B. cereus* ATCC^®^33019™) and three gram-negative bacteria (*E. coli* O157:H7 IMR E91, *C. sakazakii* ATCC^®^ 25944™, and *S. typhimurium* ATCC^®^14028™). Both fermented and nonfermented milk extract during cold storage was tested against the selected pathogenic bacteria in microtiter plate assay. A 100 µL of Mueller-Hinton broth (MHB) containing 10^6^ CFU/mL were placed in the 96-wells plate followed by adding 100 μL of extract (10 mg/mL) in MHB, with 10% dimethyl sulfoxide (DMSO) poured into the wells. After measuring absorbance at 630 nm using an Elisa plate reader (Epoch. BioTek, Winooski, VT, USA), the plates were incubated at 37 °C for 24 h before being measured again. The positive control was MHB, with 10% DMSO with pathogenic bacteria, while the negative control was fermented and pasteurized coconut milk extract with MHB, with 10% DMSO without bacteria [12]. The results then were interpreted using the following formula:

Percentage of inhibition = (24 h control − 24 h sample)/0 h control × 100.

#### 2.7.2. Antibacterial Activity by Well Diffusion Method

The antibacterial activity of the fermented extract and nonfermented extract against a variety of pathogenic (*S. aureus* ATCC^®^25923™, *B. subtilis* ATCC^®^6633™, and *B. cereus* ATCC^®^33019™) and three gram-negative bacteria (*E. coli* O157:H7 IMR E91, *C. sakazakii* ATCC^®^ 25944™, and *S. typhimurium* ATCC^®^14028™) was determined using well diffusion assay, following the previous method [3]. Wells of 6 mm diameter were punched in the MHA plate and filled with 100 µL of the fermented and nonfermented extract. After the extract, it was to be absorbed into the agar wells. The pathogenic bacteria in Mueller-Hinton broth (MHB) containing 10^6^ CFU/mL were speared on the surface of agar using cotton swap, and methanol was used as the control. The plates were incubated at 37 °C for 24 h. The antibacterial activity of the extract against the tested bacteria were established by measuring the inhibition zone diameters (mm) around the well. All the experiments were performed in triplicate.

### 2.8. Antioxidant Activity of Fermented Coconut Milk

#### 2.8.1. DPPH Radical Scavenging Activity Assay

The radical scavenging activity was determined by the previously described method [12] with some modifications. Briefly, 100 µL of DPPH (5.9 mg in 100 mL of ethanol) was added to 50 µL of the extract (5 mg/mL) in a 96-well microtiter plate. The mixture was then shaken and incubated in a dark chamber at room temperature for 30 min. Absorbance was measured at 517 nm. The control was methanol and DPPH solution. The scavenging activity was determined according to the following equation:

DPPH activity % = (A_517_ Control − A_517_ Sample)/A_517_ Control × 100

#### 2.8.2. Ferric Reducing Antioxidant Power (FRAP) Assay

The determination of FRAP was carried out according to the previous method [13] with modification. FRAP reagent was prepared using 300 mmol/L acetate buffer pH 3.6, 10 mmol/L TPTZ (2, 4, 6- tripyridyl-s-triazine) in 40 mmol/L HCl, and 20 mmol/L FeCl_3_ 6H_2_O solution in the ratio of 10:1:1 to give the working reagent. A total of 20 μL of the extract (5 mg/mL) was mixed with 200 μL FRAP solution in the 96-well microtiter plate and incubated for 45 min at room temperature in a dark room. The absorbance was measured at 595 nm wavelength using a spectrophotometer (SPECTROstar^NANO^, BMG LabTech, Ortenberg, Germany). Known concentrations of Trolox were used to prepare standard curve with linear regression, which was used to compare the extracts. Results were expressed as mmol of Trolox equivalents per g of dry sample (mmol TE/g DW).

### 2.9. NMR Measurement and Data Preprocessing

The metabolites’ variation during fermentation and cold storage was investigated according to the previous method [8]. In Eppendorf tubes, 5 mg of each freeze-dried extract were mixed with 650 µL of DMSO-d6, containing 0.1% trimethylsilyl propionic acid (TSP). At room temperature, the mixture was vortexed for 1 min and ultrasonicated for 15 min. After that, the mixture was centrifuged for 10 min at 13,000 rpm. A supernatant volume of 550 µL was transferred to an NMR tube and submitted for NMR analysis. NMR spectra were collected using a 500 MHz Varian INOVA NMR spectrometer (Varian Inc., Palo Alto, CA, USA) set to 499.887 MHz at room temperature (25 °C) with an internal lock of DMSO-d6. To minimize water (H_2_O) signals, the presaturation (PRESAT) pulse sequence was used on all samples, and the collection duration for each spectrum was 3.54 min with 64 scans. The J-resolved spectrum was recorded in 50 min 18 s (i.e., 8 scans per 128 increments for the spin-spin coupling constant axis with spectral widths of 66 Hz and 8 K for the chemical shift axis with spectral widths of 5000 Hz), and the relaxation delay was 1.5 s. Chenomx software (version 8.2, Alberta, Canada) was used for automatically phasing and baseline adjustments on all sample spectra with a reliable setting. The ^1^H NMR spectra were also binned with the Chenomx software. All the spectra were automatically binned to ASCII files with the same parameters (0.04 spectral bin), generating an area between 0.5 and 10.0 ppm. The chemical shift range of (4.50–5.0 ppm), which is related to the water signal, was omitted, and the total variables of 222 chemical shifts were generated for each of the ^1^H NMR spectra.

### 2.10. Statistical Analyses

All trials were carried out in five replicates (*n* = 5), and data are reported as mean standard deviation. Minitab was used to perform one-way analysis of variance (ANOVA) to establish statistical significance (version 17). The Tukey’s test was used to determine significant differences between means, and values with *p* < 0.05 were considered significant. After binning the NMR spectra by Chenomx, SIMCA-P software (v. 14.0, Umetrics, Umeå, Sweden) was used to perform multivariate data analysis (MVDA) using principal component analysis (PCA) and partial least square (PLS) regression by the Parreto scaling method. The NMR chemical shifts were the variable, and the sample names were the observations in the data matrix that was generated. The heat map and Pearson test for correlation analysis among all the metabolites as well as the variable importance in the projection (VIP) showing the significant metabolites were performed using MetaboAnalyst 5.0, which is freely available on the online metabolomics analysis software (http://www.metaboanalyst.ca, accessed on 15 July 2022).

## 3. Results and Discussion

### 3.1. pH and Total Titratable Acidity (TTA) during Cold Storage

To evaluate the fermentation properties of fermented coconut milk compared to pasteurized coconut milk, the pH and total titratable acidity (TTA) were evaluated throughout cold storage (Table 1). A gradual decrease in pH values and increase in titratable acidity values were observed in the fermented coconut milk during cold storage. The pH of fermented milk decreased from 4.26 to 3.92 on the 28th day during cold storage with a significant difference (*p* < 0.05). According to this finding and the previous research, the pH of commercially fermented milk is between 3.9 and 4.2 [14]. In addition, the pH of pasteurized milk slightly decreases from 6.04 to 5.09 until the 28th day during cold storage. The longer fermented coconut milk was preserved, the lower its pH value became due to the activity of the LAB utilized in its preparation.

Similarly, the average pH of coconut milk-based yoghurt was about 4.54, owing to the action of probiotic culture [15]. In addition, a study found a significant decrease in the pH of coconut milk upon fermentation [16]. The pH value of fermented milk decreased during cold storage from 4.69 to 4.04 for 28 days. Furthermore, *Lb. casei* has the ability to produce organic acids in yogurt, causing a decrease in the pH from 4.27 to 4.03 after 60 days of cold storage [16]. Additionally, according to a number of studies, the pH value of fermented milk and pasteurized milk is highly correlated with cold storage duration. Fauziah et al. [17] found that prolonged cold storage of pasteurized milk in a refrigerator led to a decrease in pH. At 9 days of cold storage, the pH was at its lowest due to an increase in lactic acid production by acid-forming bacteria, which caused the pH to decrease. To summarize, the pH of fermented milk varies during cold storage based on the initial pH level, storage temperature and duration, and probiotic culture activity.

The acidity of fermented and pasteurized milk was determined by using the titratable acidity value. The total acidity of fermented milk ranges from 0.7 to 1.1, whereas that of pasteurized milk ranges from 0.15 to 0.41, with a significant difference (*p* < 0.05). The International Dairy Federation has recommended that the minimum value of acidity in yogurt is 0.70% [18]. A decrease in pH causes an increase in titratable acid during cold storage due to the ability of LAB to utilize carbohydrates to produce lactic acid [19]. A rise in titratable acid with a fall in pH was observed throughout the 28-day cold storage of fermented milk (0.7 to 0.9). However, it was reported that the taste of fermented milk is acceptable when the titratable acidity is maintained at 70–110 T [20]. Our findings are comparable with the findings that reported a substantial difference in the titratable acidity value of fermented soymilk before and after cold storage, which was 36.02 T to 77.50 T, respectively [21]. In contrast, during the cold storage of soy probiotic yoghurt at 4 °C, no change was observed in the pH or titratable acid value of the fermented products due to lower activity of probiotic culture during refrigerated storage [22]. Table 2 shows how the acidity of pasteurized milk changes during cold storage period at 4 °C. Fauziah et al. [17] reported that the acidity of pasteurized milk decreased significantly from the first day of storage from 0.15 to 0.38. The acidity of pasteurized milk after 9 days of cold storage ranges from 0.12 to 0.15. The LAB still alive after pasteurization cause an increase in acid production. Therefore, the storage period has an effect on the bacteria population in milk, resulting in increased lactic acid formation. According to Korean Food Standards Codex, the acidity value of milk should be less than 0.18%.

### 3.2. Microbial Analysis during Cold Storage

The microbiological quality is essential for determining the quality of the food and protecting customers from any health risks caused by microorganisms [2]. Thus, it is essential for the dairy sector to preserve live bacteria in its final products. The results of the microbial analysis are shown in Table 1. Figure S1 depicts the usage of selective media (MRS gar) with white colony appearance for enumeration of LAB. The viable cell counts of LAB in fermented coconut milk significantly increased (*p* ≤ 0.05) during the fermentation and storage period (1 to 14 days), reaching 6.4 × 10^8^ CFU/mL, and then decreased significantly after 14 days, reaching 1.6 × 10^8^ CFU/mL at 28 days. Similarly, Han et al. [16] found that the *S. salivarius* ATCC 13419 had a higher survival growth rate, reaching 13.66 CFU/mL during fermentation of coconut milk. However, no changes were found in the quantity of LAB in coconut yoghurt over 14 days of storage at 4 °C [7]. The increase in viable cells in probiotics bacteria during fermentation and cold storage indicated that the coconut milk is rich in nutrients such as sugar, fat, protein, and minerals, which support the growth of the probiotics. On the other hand, the increase in probiotic population in fermented milk that was observed during cold storage is possibly related to an increase in the acidity of the fermented milk and to the presence of oxygen [23]. A decrease in the number of *L. bulgaricus* was detected from 6.11–2.27 log CFU/mL and 9.85–3.59 CFU/mL in whole-fat (WFY) and reduced-fat coconut yoghurt (RFY), respectively, for 28 days of cold storage. After 14 days of storage, the total viable count of LAB and pH declined dramatically (Table 1). The rise in acidity and reduction in refrigeration temperature generated a harsh environment that inhibited the development of LAB. The large reduction of the probiotic culture in *Lycium barbarum* yoghurt toward the end of cold storage might be attributable to an increase in the generation of hydrogen peroxide and lactic acid and a decrease in lactose level, which is a primary source of energy for LAB [24]. This could result in a decrease in the growth rate of the probiotic culture. This is consistent with an earlier finding that LAB counts in Bambara yoghurt decrease after refrigerated storage [25]. Furthermore, several studies have shown that the pH value of fermented milk and pasteurized milk is strongly linked to the duration of cold storage. Changes in the pH of pasteurized milk during cold storage are possibly due to the buildup of metabolites such as organic acid generated by microorganisms that were naturally present in milk before pasteurization [26].

The total viable count of LAB in fermented coconut milk obtained in this study was within the acceptable standard, with the minimum number more than 10^6^ cfu/mL. The minimum number of probiotic bacteria 10^7^ CFU/mL in fermented food has been strongly recommended to provide health benefits during consumption. However, the LAB were absent in pasteurized coconut milk at 1 day and then increased significantly (*p* ≤ 0.05) after 7 days, reaching 6.2 × 10^4^ CFU/mL during cold storage at 28 days. The longer the duration of cold storage, the greater the number of bacteria resistant to pasteurization temperatures and able to live at cooling temperatures [26]. This study indicated that the pasteurization temperature reduces the amount of naturally occurring LAB in coconut milk. The number of LAB organisms that survive pasteurization increases with the length of time milk is stored, which may have an impact on the milk’s quality [27]. The pasteurization may lower the amount of LAB in milk by 2.2 log CFU/mL after one day of cold storage, with the level increasing to 7.92 log CFU/mL after 21 days of cold storage [27].

Molds and yeasts are responsible for the spoiling of fermented milk during cold storage since they can live in acidic yoghurt and at low temperatures. Table 1 shows the microbial analysis of yeast and mold for fermented and nonfermented coconut milk. Figure S2 shows the media (DRBC agar) with white colony appearance for molds and pink colony for yeast, as shown in (Figure S2). The total yeast and mold count were not discovered in pasteurized milk in this study. The high-pressure processing (HPP) and pasteurization caused fully inactivated yeast and mold in cow and goat milk [26]. In addition, molds and yeast can cause spoilage and quality deterioration of coconut milk at low pH. This discovery contradicts our pH result of pasteurized milk during cold storage. Yeast and molds in fermented coconut milk were not detected on the zero, 7^th^, or 14^th^ day during cold storage but were detected and significantly increased (*p* ≤ 0.05) on the 21^st^ and 28^th^ day of storage, with levels that ranged from 1.7 × 10^2^ to 1.2 × 10^4^ CFU/mL, respectively. This finding is consistent with the previous findings [28], which discovered a rise in yeast and molds in plant-based yoghurt after storage. Similarly, the total yeast and molds in soy yogurt showed an increase during cold storage, reaching 5.9 log CFU/mL [29]. An increase in the viable count of yeast and molds in fermented milk and yogurt during cold storage is related to an increase in acidity or decrease in potential oxygen during the fermentation process, which provide suitable conditions for yeast and molds growth [4].

The high yeast and mold counts causing spoilage could be attributed to poor cleaning practices, air incorporation, unhygienic practices, and ingredient contaminations. Further, postcontamination occurred during processing, storage, and transportation [30]. According to our results, the acceptable level of yeast and molds in fermented coconut milk were achieved during the 21^st^ day of cold storage. The acceptable value of mold and yeast in beverages and yogurt should be between 10^2^–10^3^ CFU/mL; exceeding this range cause potential health hazards and imminent spoilage due to the ability of yeast and mold to produce toxic compounds including mycotoxin, e.g., aflatoxin. However, changes in flavor, texture, taste, and discoloration occurred during the growth of yeast and molds more than the acceptable level [29].

Coliform bacteria are gram-negative bacteria, such as *Escherichia coli,* which are often used to test the quality of milk. This category of bacteria may create acid and gas through lactose fermentation with or without oxygen [31]. To enumerate coliform and *E. coli*, we used selective media (coliform agar) with pink colony appearance for coliform and blue colony for *E. coli* (Figure S3). As stated in (Table 1), no growth of coliforms or *E. coli* was seen in coconut milk on the first and seventh days. However, the growth of coliform and *E. coli* significantly increased (*p* ≤ 0.05) and was observed in the 14^th^ until the 28^th^ day of cold storage, recorded as 2.7 × 10^1^, 3.7 × 10^2^, 4.7 × 10^2^, 1.4 × 10^1^, 2.6 × 10^2^, and 3.4 × 10^2^ CFU/mL for coliform and *E. coli* during the 14^th^, 21^st^, and 28^th^ day, respectively. Similarly, a rise in coliform count during the 6^th^ day of refrigerated storage reaching 3.72 (±0.17) log CFU/mL was observed [32]. Certain coliform and *E. coli* bacteria, as well as other heat-resistant gram-negative bacteria that enter milk after pasteurization, may reach spoiling levels in refrigerated storage as soon as the seventh day after pasteurization [32]. The presence of coliforms indicates fecal contamination and a poor level of hygiene after processing [32]. In addition, a high level of these bacteria is an indicator of the inefficacy of hand washing and contamination of raw foods, equipment, and the place where food is prepared [31]. Sanitation and cleaning programs can reduce this type of pathogenic bacteria and extend the shelf life of milk up to the 17^th^ day [33]. According to the standards of GSO 1016, pasteurized milk produced in 2015 shall be free of *E. coli* colonies [34]. However, according to Turkish Standard Institute (TSI330), milk products should contain no more than 10 coliform colonies [35].

The coliform and *E. coli* test gave negative results, and no growth was observed in fermented coconut milk during the 28^th^ day of cold storage (Table 1). This is consistent with [28] investigation, which found no Salmonella, coliform, *E. coli*, or fecal enterococci in plain yoghurt. Similarly, no growth for coliform or *E. coli* was observed in soy yogurt during cold storage [21]. In addition, mung bean-enriched stirred yoghurt was coliform-free after 28 days of cold storage [36]. There was an absence of coliform and *E. coli* in fermented milk and yogurt due to the ability of probiotic bacteria to produce organic acid, hydrogen peroxide, and secondary metabolites such as bacteriocin that can inhibit the growth of pathogenic bacteria. In addition, the refrigeration temperature and acidic environment create undesirable and harsh conditions for coliform growth [37]. According to our results, we conclude that the fermentation extended the shelf life of coconut milk to 21 days under refrigeration condition compared to fresh coconut milk with 7 days of shelf life under refrigeration condition.

### 3.3. Proximate Analysis of Fermented Coconut Milk and Fresh Coconut Milk

The predominant constituents of nonfermented and fermented coconut milk were fat, followed by protein and carbohydrates. The effect of fermentation on the proximate composition of fermenting coconut milk is presented in Table 2. Proximate composition for coconut milk is recorded as total fat (19.27%), carbohydrate (4.13%), energy (197.3%), protein (1.86%), moisture (74.05%), total ash (0.68%), and total solid (25.95%). These compositions in fermented coconut milk were 19.37, 2.51, 193.3, 2.31,75.14, 0.66, and 24.85%, respectively. After fermentation, ash, total solid carbohydrate, and energy content decreased, while protein and moisture content increased; no change in fat content was found. The proximate composition of coconut milk is ash (0.52%), moisture (65.00%), fat (15.02%), and crude protein (7.17%) [38]. The proximate composition of Malaysian coconut milk contains fat (15.44%), protein (3.40%), moisture (73.57%), and ash (0.71%). In contrast to our results, the fat content of soymilk decreases after natural fermentation due to the action of lipolytic enzymes, which hydrolyze fat components [39]. In addition, the fermentation process is ascribed to bacteria-producing enzymes that are classified as protein and break down these molecules. During fermentation, this causes an increase in protein content. However, *L. plantarum* may create proteinaceous enzymes during fermentation, boosting the protein concentration [40]. Furthermore, the increased protein content of coconut milk after fermentation might be related to the production of various substances during fermentation, such as peptides and amino acids, which are considered protein [41].

The carbohydrate content of coconut milk decreases from 4.13 to 2.51% during fermentation. It was reported that the bacteria used glucose as a source of energy during fermentation, which explains the decline in carbohydrates after fermentation [40,41]. The moisture percentage was somewhat higher than that previously reported [7], which found out that the moisture content of coconut-flavored yoghurt was about 71.31%. The quantity of moisture content in our research rose somewhat after fermentation, which agrees with prior evidence that a rise in moisture content during fermentation is attributable to the generation of a little amount of water [42]. A decrease in moisture content during fermentation was possibly due to the increase in the formation of dry matter content [39].

The protein content of fermented coconut milk increased from 1.86% to 2.3% after fermentation. Our findings are consistent with the previous study, which demonstrated that the protein content of yoghurt rose as fermentation duration increased [43]. Furthermore, another study revealed that the fermentation duration had a substantial effect on the protein content of horse gramme flour during fermentation [44]. The rise in protein concentration during fermentation may be ascribed to the creation of protein-based enzymes by bacteria, as well as the breakdown of certain molecules. Furthermore, *L. plantarum* may create proteinaceous enzymes during fermentation, hence boosting the protein content [40]. Additionally, the increase in protein content of coconut milk after fermentation might be related to the production of peptides and amino acids during fermentation [40].

### 3.4. Antibacterial Activity of Fermented and Nonfermented Coconut Milk

**Figure 1:**
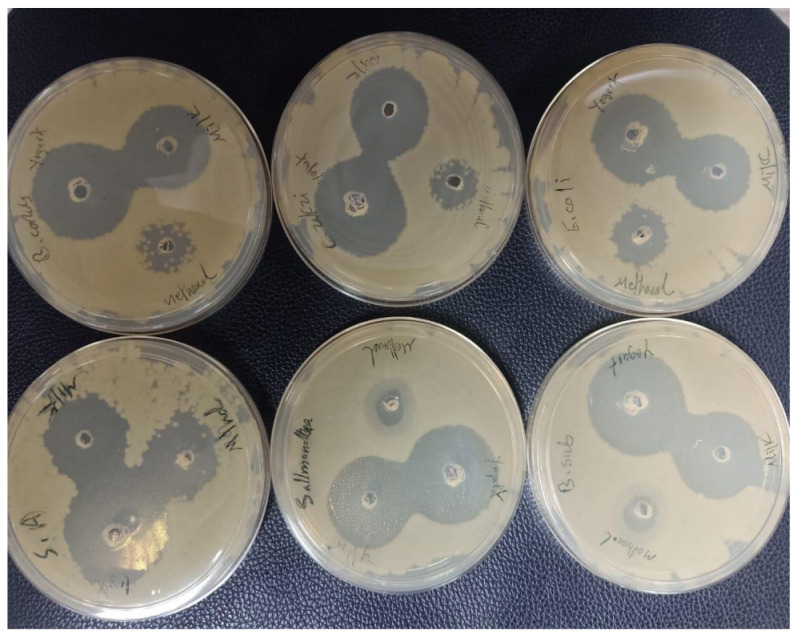
Plate showing inhibition zone of extract against target bacteria.

The antibacterial activity of fermented and pasteurized extracts was studied during 21st and 7th cold storage, respectively, against three gram-positive bacterial strains (*S. aureus* ATCC^®^25923™, *B. subtilis* ATCC^®^6633™, and *B. cereus* ATCC^®^33019™) and three gram-negative bacteria strains (*E. coli* 157:H7 IMR E9, *C. sakazakii* ATCC^®^ 25944™, and *S. typhimurium* ATCC^®^14028™) using the well diffusion technique and microtiter plate assay, as shown in Table 3 and Table 4. The microtiter plate assay was used to measure the absorbance at 630 nm using Elisa plate reader at 0 and 24 h. The well diffusion method of the antibacterial was used to measure the zone inhibition around the well (Figure 1). The microtiter plate assay results show that the highest antibacterial activity recorded in fermented coconut milk were: F14 95.3% and 94.73 against *E. coli* and *B. cereus*, respectively, F1 90.6% against *B. cereus,* F7 94.04% against *B. cereus*, and F21 88.63% against *B. subtilis.* In comparison to fermented coconut milk, pasteurized coconut milk had a modest antibacterial impact on target microorganisms. The highest antibacterial activity of nonfermented milk was at M1 45.6% against *C. sakazakii* and M7 44.93% against *C. sakazakii.* Similarly, the highest antibacterial activity recorded in fermented coconut milk are F14 with zone inhibition (23 mm) against *S. Typhi,* F7 against *E. coli* (21.33 mm), F21 against *S. typhi* (18.66 mm), and F1 against *C. sakazakii* (15.33 mm). Nonfermented milk had the lowest bactericidal activity: M1 against *C. sakazakii* and *B. subtilis* (10.66 mm), and M7 against *B. subtilis* (9.66 mm) [41].

Milk fermentation’s antimicrobial properties may be owing to LAB, which secreted organic acids, such as diacetyl, acetaldehyde, and ethanol, as well as bacteriocins [45]. In addition, kefir and probiotic bacteria consume polysaccharides, peptides, and proteins to generate organic acids and bioactive compounds that prevent the development of pathogenic microorganisms. The organic acid in nondissociated objects may penetrate the cell wall in the form of dissociated organic acid, which is followed by a decrease in intracellular pH, which leads to the destruction of the cytoplasm of pathogens.

Similarly to our study, Lakshmi et al. [46] reported that the fermented coconut milk exhibited antimicrobial and antifungal activities against *E. coli*, *S. typhi, saccharomyces cerevisiae,* and *Aspergillus niger* due to the production of organic acids, peptides (bacteriocins), carbon dioxide, and ethanol during fermentation. The fermentation process can increase and improve the antibacterial activity of fermented coconut milk in comparison to pasteurized coconut milk. The fermentation of coconut milk with *S. salivarius* showed strong antibacterial activity against *Streptococcus pyogenes* in comparison to pasteurized coconut milk [16]. Furthermore, the antibacterial peptides produced in fermented milks with specific *L. plantarum* showed activity against *S. aureus, E. coli, S. Typhimurium, S. choleraesuis* ssp. *choleraesuis serovar Choleraesuis,* and *L. innocua* [47].

Our results indicate that pasteurized coconut milk exhibited antibacterial activity against selected target bacteria. Coconut milk’s lauric acid has been shown to have antibacterial properties against pathogenic microorganisms [16,47]. In addition, during fermentation, probiotic bacteria are able to convert lauric acid to monolaurin, which has a far higher antibacterial activity than lauric acid [16]. As shown in Table 3 and Table 4, the antibacterial activity of fermented milk increased during cold storage until the 14th day, while it decreased on the 21st day toward pathogenic bacteria at the end of the cold storage period. The reduction or rise in antibacterial activity during cold storage might be attributed to antibacterial chemicals produced during fermentation and cold storage interacting with one another to enhance or reduce antimicrobial activity [47]. It was reported that the antibacterial activity of fermented milk increases during cold storage due to the accumulation of antibacterial bioactive peptide as a result from increases in the degree of proteolysis [48].

### 3.5. Antioxidant Activity of Fermented and Pasteurized Coconut Milk

The antioxidant activity of fermented coconut milk was considerably greater than that of pasteurized coconut milk in this research, as assessed by FRAP and DPPH tests (Table 5). The fermented coconut milk had the greatest DPPH and FRAP values of 67.1% and 61.961 mmol/g at day 14, whereas FCM had the lowest DPPH and FRAP values of 58% and 55.113 mmol/g at day 21. Both coconut milk and coconut oil have been shown to have greater levels of phenolic compounds in prior research [49]; coconut milk is rich in antioxidant compounds such as amino acid and vitamins E and C [8]. Fermentation is a process used to increase the nutritional content and antioxidant activity of food via the synthesis of bioactive molecules such as peptides and phenolic compounds. Moreover, pH is a significant element that might affect antioxidant activity by altering the structure and concentration of bioactive molecules [50]. *Lactobacillus* strains display proteolytic activity and release bioactive peptides with antioxidant activity from milk protein during milk fermentation [51]. It was also reported that fermented milk produced by *L. plantarum* exhibited antioxidant activity displayed at a 14.7% to 48.9% (DPPH) inhibition rate [52]. Valero-Cases and Frutos [53] determined the ability of LAB in pomegranate juices to alter and biotransform phenolic compounds into two new phenolic derivatives. Not only does the fermentation process utilizing LAB boost the bioactive molecule, but these LAB also have their own antioxidative capabilities by creating enzymatic and nonenzymatic antioxidants to defend themselves from oxidative damage [54].

The antioxidant activity of fermented coconut milk could be influence by a long cold storage period. The antioxidant activity of Labneh increases with cold storage for up to 20 days due to the proliferation of probiotic bacteria and its proteolytic enzymes secretion [55]. Antioxidant activity in fermented coconut milk rose throughout the fermentation process in this study; however, after 21 days there was a reduction, which is in line with earlier research. The bioactive component may undergo various transformations, degradation, oxidation, and hydrolysis during fermentation and cold storage. A reduction in antioxidant activity may be caused by the oxidation of phenolic compounds as a consequence of dissolved oxygen in fermented cornelian cherry juice with *L. casei* T4 [56]. Similarly, Kurnia et al. [57] reported that the antioxidant activity of fermented goat milks using *L. fermentum* PE2 decreases during cold storage due to damage to the structure of the bioactive compounds. Therefore, fermentation of coconut milk with *L. plantarum* ngue16 can improve and enhance the antioxidant activity in comparison to the nonfermented milk.

### 3.6. Bioactive Metabolites of Fermented Coconut Milk

The discovered metabolites in fermented and pasteurized coconut milk were varied, with components detected spanning from amino acids to fatty acids, carbohydrates, and chemical molecules (Table 6 and Figure S4).

**Figure 2:**
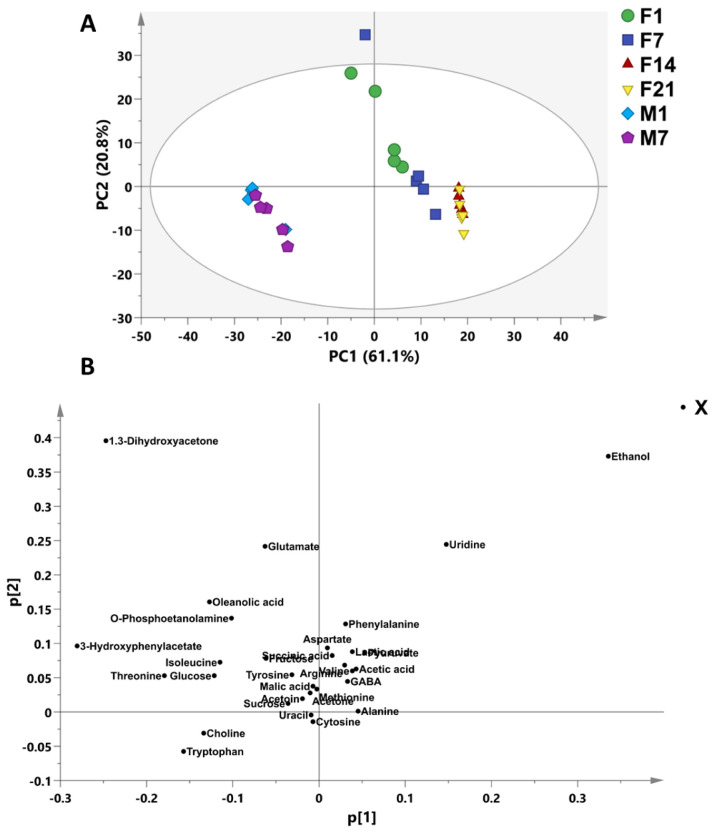
The PCA score (**A**) and loading (**B**) plot of fermented coconut milk and pasteurized coconut milk. M1-M7 (pasteurized coconut milk during 7-day cold storage). F1-F21 (fermented coconut milk during 21 days of cold storage), M1-M7 (coconut milk during 7-day cold storage).

The variation in metabolite contents of fermented coconut milk and pasteurized coconut milk samples extracted during cold storage were assessed using multivariate data analysis (MVDA). The principal component analysis (PCA) was applied to understand the clustering features of the samples and the metabolites that provided the variability. The PCA score plot showed the clustering of the samples, and loading plot indicated that the variable contributed to the sample differences (Figure 2A). As shown in the PCA score plot, the first principal component (PC1) accounted for 61.1% of the variation in the data, whereas PC2 was able to explain 20.8% of the variation (Figure 2A). The score plot (Figure 2B) revealed that there are two clear clusters, which are fermented coconut milk and pasteurized coconut milk. The metabolites responsible for this difference were sucrose, glucose, fructose, including ethanol, valine, gamma-aminobutyric acid (GABA), arginine, lactic acid, acetoin, alanine, phenylalanine, acetic acid, methionine, acetone, pyruvate, succinic acid, malic acid, tryptophan, uridine, uracil, and cytosin (Figure 2B). This result indicates that the fermentation and cold storage time could contribute to the metabolites’ variation. The major metabolites observed were ethanol, lactic acid, GABA, acetic acid, pyruvate, and uridine, which increased due to the fermentation with LAB. In addition, acetate, acetoin, and different amino acids were found at different concentrations in the fermented coconut milk. The concentration of the three main sugars in coconut milk, glucose, sucrose, and fructose, were in decline after the fermentation. The lactic acid bacteria utilize mono- and disaccharides during fermentation to produce mainly lactic acid, acetic acid, and several other acids [58] as well as an increase in acetone and ethanol as volatile compounds during fermentation [59]. In addition, the fermentation process reported to enhance and produce essential amino acids during fermentation. Proteolytic activity of *L. lactis* used in food production as a starter culture due to the ability of some *L. lactis* strains to generate peptides and amino acids during fermentation such as isoleucine, leucine, valine, histidine, and methionine [60]. However, Das et al. [61] reported that *L. plantarum* NRRL B-4496 isolated from fermented beverage has the ability to utilize monosodium l-glutamate to produce bioactive GABA through glutamate decarboxylase enzyme (GAD).

**Figure 3:**
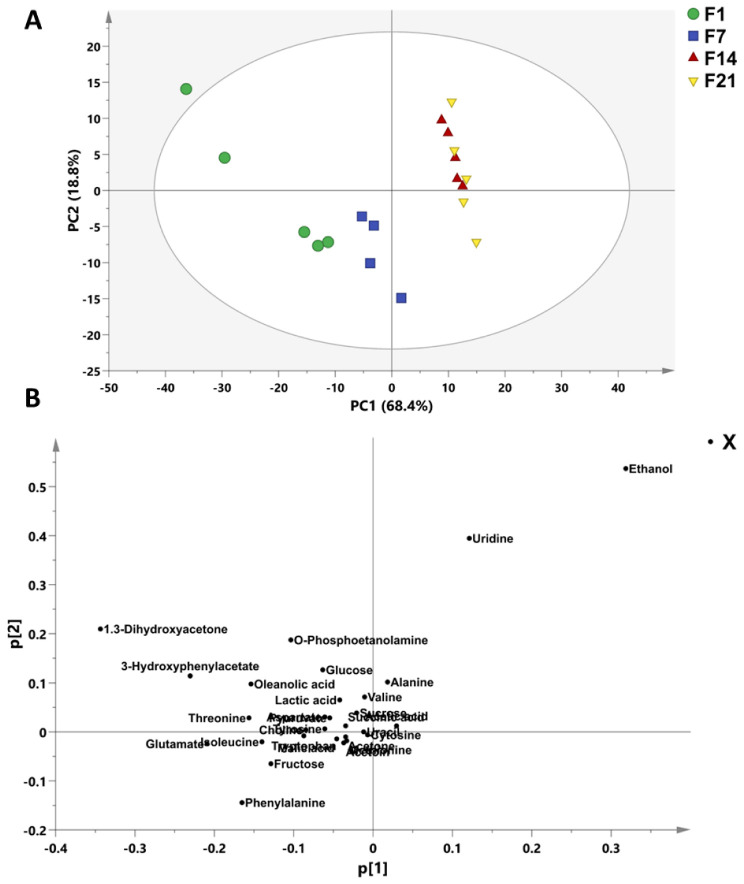
The PCA score (**A**) and loading (**B**) plot of fermented coconut milk during cold storage. F1–F21 (fermented coconut milk during 21-day cold storage).

**Figure 4:**
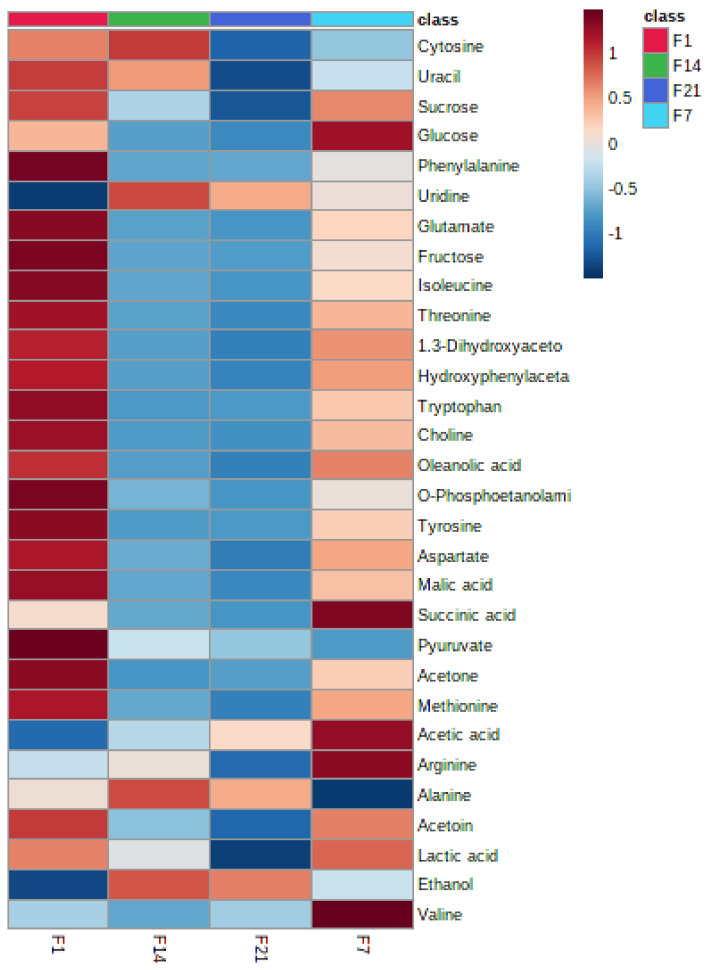
The heatmap showing the metabolites’ differences of fermented coconut milk during cold storage. F1-F21 (fermented coconut milk during 21-day cold storage). Color of the squares are associated with the differences. High contribution in the variation is shown in red (dark red with the strongest contribution), whereas low contribution is shown in blue (dark blue with the weakest contribution).

To observe the variation among fermented coconut milk during cold storage at 1, 7, 14, and 21 days, other models were generated. The processed ^1^H NMR data of fermented coconut milk during cold storage at 1, 7, 14, and 21 days were subjected to PCA, and the results are shown in Figure 3. As shown in the PCA score plot, the first principal component (PC1) accounted for 68.4% of the variation in the data, whereas PC2 was able to explain 18.8% of the variation (Figure 3A). The fermented coconut milk extracts during cold storage were separated into two clusters. The fermented coconut milk at 1 day were well separated from samples at 14 and 21 days. The samples at 14 and 21 days were similar, and a slight difference among their metabolites was observed. The fermented coconut milk at 7 days of storage was a typical group that has most metabolites. The loading plot (Figure 3B) shows the metabolites responsible for this variation, including alanine, ethanol, o-Phosphoetanolamine, valine, GABA, succinic acid, acetic acid, tyrosine, and uridine in 14 and 21 days during cold storage. However, 1.3-dihydroxyacetone, 3-hydroxyphenylacetate, o-Phosphoetanolamine, oleanolic acid, threonine, glucose, choline, lactic acid, glutamate, Isoleucine, fructose, and phenylalanine were higher in fermented milk at the first day of storage (Figure 4). The result indicates that the fermentation process as well as the cold storage could affect the metabolites’ variation during cold storage of fermented coconut milk. The fermentation and cold storage cause an increase in titratable acidity due to the availability of sugar that is utilized by LAB to produce organic compounds, which can contribute to metabolites’ activity of LAB. Additionally, the fermentation participated in an increase in metabolites formation [25,60]. Baba et al. [62] reported that the higher proteolytic activity of the *L. barbarum* in yogurt on day 7 during cold storage was due to the ability of *L. barbarum* to release an amino acid through the activity of proteinase and peptidase enzymes.

**Figure 5:**
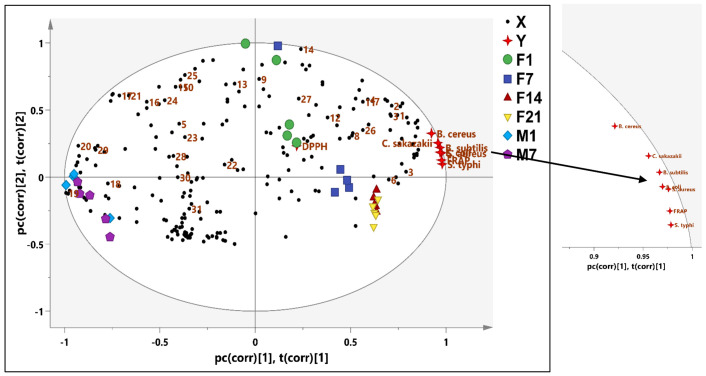
The biplot obtained from PLS describing the correlation between the metabolites with antioxidant and antibacterial activities of fermented and pasteurized coconut milk. M1–M7 (pasteurized coconut milk during 7-day cold storage). F1–F21 (fermented coconut milk during 21-day cold storage). GABA (1), valine (2), ethanol (3), lactic acid (4), acetoin (5), alanine (6), arginine (7), acetic acid (8), methionine (9), acetone (10), pyruvate (11), succinic acid (12), malic acid (13), aspartate (14), tyrosine (15), O-phosphoetanolamine (16), oleanolic acid (17), choline (18), tryptophan (19), 3-hydroxyphenylacetate (20), 1.3-dihydroxyacetone (21), threonine (22), isoleucine (23), fructose (24), glutamate (25), uridine (26), phenylalanine (27), glucose (28), sucrose (29), uracil (30), and cytosine (31). DPPH; 1,1-diphenyl-2-picrylhydrazyl, FRAP; ferric reducing antioxidant power.

**Figure 6:**
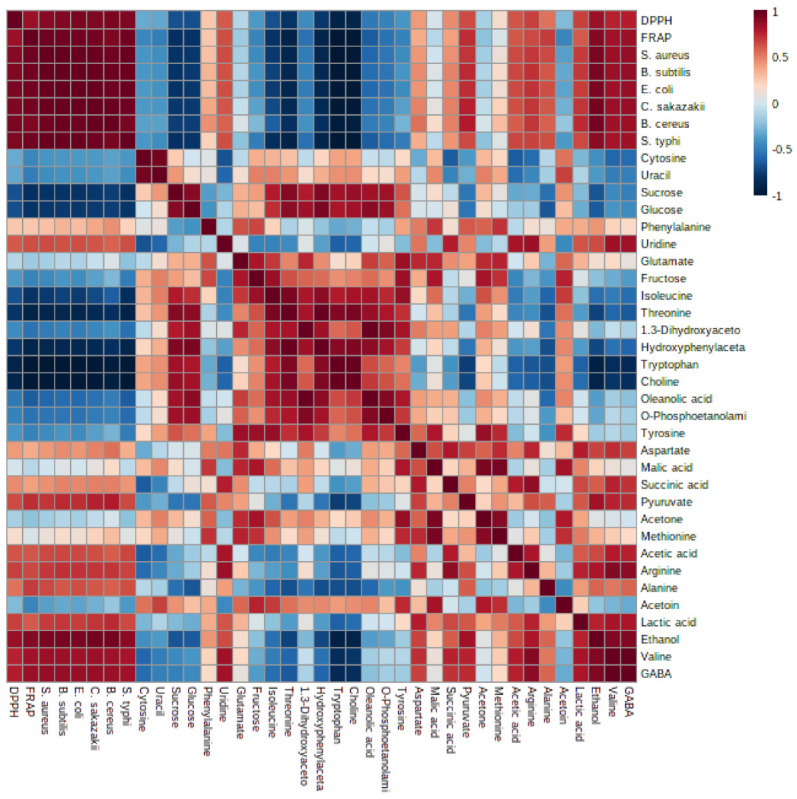
The Pearson’s correlation of the most significant metabolites obtained from PLS model describing the correlation between the metabolites with antioxidant and antibacterial activities of fermented and pasteurized coconut milk. Color of the squares are proportional to the correlation coefficients. Positive correlations are shown in red (dark red with the strongest correlation), whereas negative correlations are in blue (dark blue with the weakest correlation). DPPH; 1,1-diphenyl-2-picrylhydrazyl, FRAP; ferric reducing antioxidant power.

Few studies mentioned that the coconut milk, especially when fermented, provides health benefits with different biological activities. However, there has been no information on the metabolites profile of the fermented coconut milk. In this study, metabolomics was performed to correlate antibacterial and antioxidant activity of the fermented coconut milk and pasteurized coconut milk with their metabolites profile. In this study, the PLS model demonstrated the significant correlation between the metabolites of the fermented coconut milk and antioxidant (DPPH and FRAP) and antibacterial activity (*S. aureus, B. subtilis, E. coli, C. sakazakii, B. cereus* and *S. typhi*) (Figure 5). It can be observed that the fermented coconut milk was correlated more toward the antibacterial and antioxidant activity. The compounds of the fermented coconut milk contributing to the antioxidant and antibacterial activity to the pathogenic bacteria were ethanol, GABA, uridine, valine, lactic acid, alanine, arginine, acetic acid, methionine, acetone, pyruvate, succinic acid, malic acid, aspartate, threonine, and phenylalanine. Some of these metabolites have previously been investigated as antibacterial agents against pathogenic bacteria. As shown by the correlation coefficients in Figure 6, acetic acid and alanine have been reported to show strong antimicrobial activity against *B. cereus, B. subtilis, B. megaterium*, and *B. pumilus* [8]. These identified compounds were suggested to be responsible for the biological activities of fermented foods, which is in agreement with our study [63]. The Pearson correlation supports the PLS data. Bioactive metabolites with known antimicrobial activity against *E. coli, S. typhimurium, Aspergillus flavus*, and *Penicillium* spp. were identified in fermented cantaloupe juice, such as lactic acid, GABA and beta-alanine [63], ethanol, and organic acid against *S. Arizonae* and *S. Typhimurium* [64]. However, acetic, malic, lactic, fumaric, benzoic, sorbic acids, sulfite, and succinic acid had the ability to inactivate the growth acid-resistant vegetative pathogens *E. coli* O157:H7 and *S. aureus* [63,64]. The antioxidant activity of the metabolites arginine and GABA was reported [65,66]. In addition, uridine identified in black garlic exhibited strong antioxidant activity [67]. The identified α-linolenic acid, g-oryzanol, a-tocopherol, GABA, a-aminobutyric acid, glutamic acid, leucine, hydroxyl-L-proline, 3-hydroxybutyric acid, 2,3- butanediol, fumaric acid, fatty acids, vanillic acid, phenylalanine, and valine were associated with antioxidant activity of germinated rice [68]. The fermentation process is useful as a natural preservation method of food with antibacterial and antioxidant activities via increasing the free total phenolic and organic acids, exopolysaccharides, bacteriocins, and bioactive peptides [69]. The outcome of this study showed that the fermentation process improved the antibacterial and antioxidant activities of the metabolites of the fermented coconut milk.

**Figure 7:**
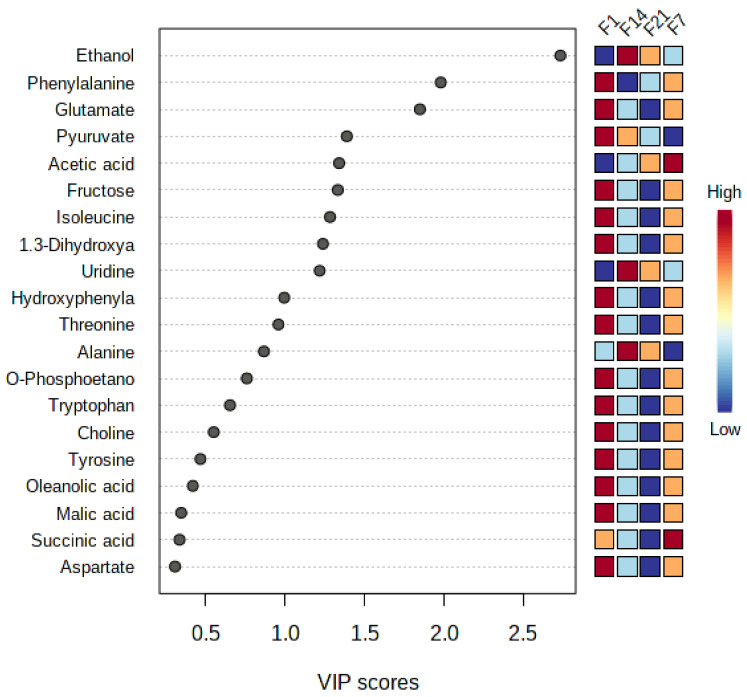
The variables important in the projection (VIP) values derived from PLS showing the significant metabolites with antioxidant and antibacterial activities of fermented and pasteurized coconut milk. M1–M7 (pasteurized coconut milk during 7-day cold storage). F1–F21 (fermented coconut milk during 21-day cold storage).

Using the PLS-derived biplots, the variable importance in projections (VIP) values were used to identify the major factors that contribute to the biological activity. The VIP value is calculated by summing the squares of the PLS weights while taking the Y variance in each dimension into account. The VIP values show how much each variable contributed to separating clusters in the PLS biplot (Figure 7). The variables with VIP values more than 0.5 higher are important and significant in the correlation and projection in the PLS model, and thus, they are associated with chemical markers and/or bioactive compounds from the fermented coconut milk [68].

In this study, the Q2 and R2 values were both greater than 0.8, indicating that all models met the validation and prediction performance standards. The 100 permutation tests and regression validation demonstrated that the PLS model was valid. To determine and validate the relationship between the variables, correlation coefficients (R) were determined. The experimental values of the bioactivities were derived as regression plots as a function of the predicted values for sample validation. The experimental values of the bioactivities were derived as a regression plot (Figure S5) as a function of the predicted values for sample validation. These data also suggested that PLS models were useful for predicting and validating the parameters.

## 4. Conclusions

The study reports on the potency of developing probiotic milk with potent biological activity using coconut milk and the fermentation process with *L. plantarum* ngue16. The fermentation for 15 h at 33 °C significantly extended the shelf life and improved the antibacterial and antioxidant activities of coconut milk compared to pasteurized coconut milk. The fermented coconut milk demonstrated low microbial load and stable shelf life for 21 days at 4 °C. The biological activity of fermented coconut milk was due to the presence of several bioactive compounds, including ethanol, GABA, uridine, valine, lactic acid, alanine, arginine, acetic acid, methionine, acetone, pyruvate, succinic acid, malic acid, aspartate, threonine, and phenylalanine, which were identified using ^1^H NMR. The results of the study demonstrated the high potential for *L. plantarum* ngue16 to develop fermented coconut milk with enhanced antibacterial and antioxidant activities and expand the shelf life. Further study is recommended to optimize the fermentation conditions to enhance the bioactive compounds in the fermented coconut milk.

## Figures and Tables

**Table 1:** The pH, total titratable acidity, and microbial changes of fermented and pasteurized coconut milk during cold storage.

Parameters	Fermented Coconut Milk					Pasteurized Coconut Milk				
	Days of Storage									
	1	7	14	21	28	1	7	14	21	28
pH	4.26 ^a^ ± 0.10	4.20 ^a^ ± 0.11	4.17 ^a^ ± 0.12	4.13 ^a^ ± 0.32	3.92 ^b^ ± 0.02	6.04 ^c^ ± 0.08	5.92 ^c^ ± 0.07	5.65 ^d^ ± 0.05	5.39 ^e^ ± 0.03	5.09 ^f^ ± 0.04
Acidity	0.7 ^a^ ± 0.02	0.82 ^b^ ± 0.02	0.92 ^b^ ± 0.01	0.93 ^c^ ± 0.02	1.1 ^d^ ± 0.02	0.15 ^d^ ± 0.005	0.22 ^e^ ± 0.03	0.32 ^ef^ ± 0.02	0.35 ^f^ ± 0.01	0.41 ^g^ ± 0.03
Lactic acid bacteria	3.43 × 10^8 a^ ± 0.41	5.26 × 10^8 b^ ± 0.25	6.4 × 10^8 c^ ± 0.49	2.1 × 10^8 d^ ± 0.26	1.6 × 10^8 e^ ± 0.15	Absent ≤1.0 × 10^1^	4.6 × 10^2 f^ ± 0.17	4.3 × 10^3 g^ ± 0.25	3.1 × 10^4 h^ ± 0.20	6.2 × 10 ^4 i^ ± 0.25
Total yeast and mold	Absent ≤1.0 × 10^1^	Absent ≤1.0 × 10^1^	Absent ≤1.0 × 10^1^	1.7 × 10^2 a^ ±0.30	1.2 × 10^4 b^ ±0.15	Absent ≤1.0 × 10^1^	Absent ≤1.0 × 10^1^	Absent ≤1.0 × 10^1^	Absent ≤1.0 × 10^1^	Absent ≤1.0 × 10^1^
Coliform	Absent ≤1.0 × 10^1^	Absent ≤1.0 × 10^1^	Absent ≤1.0 × 10^1^	Absent ≤1.0 × 10^1^	Absent ≤1.0 × 10^1^	Absent ≤1.0 × 10^1^	Absent ≤1.0 × 10^1^	2.7 × 10^1 a^ ± 0.15	3.7 × 10^2 b^ ± 0.28	4.7 × 10^2 c^ ± 0.35
*E. coli*	Absent ≤1.0 × 10^1^	Absent ≤1.0 × 10^1^	Absent ≤1.0 × 10^1^	Absent ≤1.0 × 10^1^	Absent ≤1.0 × 10^1^	Absent ≤1.0 × 10^1^	Absent ≤1.0 × 10^1^	1.4 × 10^1^± 0.28	2.6 × 10^2^ ± 0.30	3.4 × 10^2^ ± 0.20

Values are expressed as mean ± standard deviation (*n* = 5). Different superscript letters represent significant differences within the row (*p* < 0.05).

**Table 2:** Proximate analysis of fermented coconut milk and pasteurized coconut milk.

Parameters	Fermented Coconut Milk (kcal/100 g)	Pasteurized Coconut Milk (kcal/100 g)
Total fat	19.37 ^a^ ± 0.11	19.27 ^a^ ± 0.05
Carbohydrate	2.51 ^a^ ± 0.025	4.13 ^b^± 0.034
Energy	193.3 ^a^ ± 0.57	197.3 ^b^ ± 0.57
Protein	2.31 ^a^ ± 0.155	1.86 ^b^ ± 0.096
Moisture	75.14 ^a^ ± 0.015	74.05 ^b^ ± 0.01
Total Ash	0.66 ^a^ ± 0.02	0.68 ^a^ ± 0.02
Total Solid	24.85 ^a^ ± 0.015	25.95 ^b^ ± 0.01

Values are expressed as mean ± standard deviation (*n* = 5). Different superscript letters represent significant differences within the row (*p* < 0.05).

**Table 3:** Antibacterial activities of methanol extract of fermented and pasteurized coconut milk by microtiter plate assay.

Samples	*S. aureus*	*B. subtilis*	*E. coli*	*C. sakazakii*	*B. cereus*	*S. Typhi*
M1	41.86 ^a^ ± 1.56	41.36 ^a^ ± 1.26	40.2 ^a^ ±1.16	45.6 ^a^ ± 0.60	41.26 ^a^ ± 1.20	40.3 ^a^ ± 0.91
M7	41.73 ^a^ ± 1.45	43.33 ^a^ ± 1.00	42.63 ^a^ ± 0.75	44.93 ^a^ ± 0.66	40.3 ^a^ ± 1.11	39.9 ^a^ ± 1.41
F1	76.93 ^b^ ± 2.07	83.93 ^b^ ± 2.12	81.66 ^b^ ± 2.15	86.63 ^b^ ± 2.70	90.06 ^b^ ± 2.66	71.9 ^b^ ± 2.45
F7	89.0 ^c^ ± 0.45	93.30 ^c^ ±1.37	90.70 ^c^ ± 2.05	92.6 ^c^ ±2.06	94.03 ^b^ ± 1.10	85.90 ^c^ ± 2.11
F14	91.13 ^c^ ±0.72	93.7 ^c^ ±1.96	95.3 ^c^ ± 1.41	93.66 ^c^ ± 1.05	94.73 ^b^ ± 2.32	89.60 ^c^ ± 2.04
F21	88.53 ^c^ ±1.40	88.63 ^bc^ ± 0.90	85.06 ^b^ ± 1.35	86.43 ^b^ ± 2.24	80.73 ^c^ ± 2.15	83.43 ^c^ ± 1.95

The values are percentage of inhibition (%) = [(Control absorbance − Sample absorbance)/Control absorbance] ×100. Values are expressed as mean ± standard deviation (*n* = 5). Different superscript letters represent significant differences within the row (*p* < 0.05). M1: pasteurized coconut milk at one day cold storage, M7: pasteurized coconut milk at 7-day cold storage, F1: fermented coconut milk at one day cold storage, F7: fermented coconut milk at 7-day cold storage, F14: fermented coconut milk at 14-day cold storage, and F21: fermented coconut milk at 21-day cold storage.

**Table 4:** Antibacterial activities of methanol extract of fermented and pasteurized coconut milk by well diffusion method.

Samples	*S. aureus*	*B. subtilis*	*E. coli*	*C. sakazakii*	*B. cereus*	*S. Typhi*
M1	9.33 ^a^ ± 0.57	10.66 ^a^ ± 1.52	9.33 ^a^ ± 0.57	10.66 ^a^ ± 0.57	8.33 ^a^ ± 1.15	8.66 ^a^ ± 1.52
M7	8.66 ^a^ ± 0.57	9.66 ^a^ ± 0.57	8.66 ^a^ ± 0.57	8.33 ^b^ ± 0.57	8.66 ^a^ ± 1.15	8.33 ^a^ ± 0.57
F1	13.33 ^b^ ± 1.52	14.33 ^b^ ± 1.15	14.0 ^b^ ± 1	15.33 ^c^ ± 1.52	13.66 ^b^ ± 1.15	14.33 ^b^ ± 0.57
F7	16.33 ^c^ ± 0.57	18.66 ^c^ ± 1.52	21.33 ^c^ ± 2.08	20.66 ^d^ ± 1.52	15.33 ^bd^ ± 1.52	14 ^b^ ± 1.73
F14	20 ^d^ ± 1	22.66 ^d^ ± 2.08	22.0 ^c^ ± 2	22.66 ^d^ ± 1.52	20.66 ^c^ ± 1.15	23 ^c^ ± 1.73
F21	15.33 ^bc^ ± 1.52	16 ^bc^ ± 1.73	14.33 ^b^ ± 2.08	14.33 ^c^ ± 1.5	17.33 ^d^ ± 1.52	18.66 ^d^ ± 1.52

The values are the zone of inhibition (mm). Values are expressed as mean ± standard deviation (*n* = 5). Different superscript letters represent significant differences within the column (*p* < 0.05). M1: pasteurized coconut milk at one day cold storage, M7: pasteurized coconut milk at 7-day cold storage, F1: fermented coconut milk at one day cold storage, F7: fermented coconut milk at 7-day cold storage, F14: fermented coconut milk at 14-day cold storage, F21: fermented coconut milk at 21-day cold storage.

**Table 5:** Antioxidant activity of the fermented and pasteurized coconut milk as determined using DPPH (%) and FRAP (mmol TE/g) assays.

Sample	DPPH (%)	FRAP (mmol TE/g)
M1	48.0 ^a^ ± 0.022	20.38 ^a^ ± 0.17
M7	46.0 ^a^ ± 0.015	17.80 ^a^ ± 0.90
F1	59 ^b^ ± 0.011	53.53 ^b^ ± 1.64
F7	65.0 ^c^ ± 0.012	56.70 ^b^ ± 2.66
F14	67.17 ^c^ ± 0.024	61.96 ^c^ ± 0.78
F21	58.0^b^ ± 0.014	55.11^b^ ± 2.68

Values are expressed as mean ± standard deviation (*n* = 5). Different superscript letters represent significant differences within the column (*p* < 0.05).

**Table 6:** ^1^H NMR signals of metabolites identified in fermented coconut milk and pasteurized coconut milk.

**Metabolites**	^1^H NMR Signals	**Fermented Coconut Milk**	Pasteurized **Coconut Milk**
Ethanol	1.22 (t, J = 7.0 Hz)	+	-
Valine	1.02 (m)	+	-
ϒ-Aminobutyric acid (GABA	1.90 (m), 0.98 (t)	+	-
Arginine	1.78 (m). 3.82 (t)	+	-
Lactic acid	1.31 (d, J = 6.5)	+	-
Acetoin	δ 1.42 (s)	+	-
Alanine	1.5 (d, J = 7.0 Hz)	+	-
Threonine	3.62 (d, J = 7.0 Hz)	-	+
Phenylalanine	3.9 (q)	+	-
Acetic acid	1.96 (s)	+	-
Methionine	2.13 (s)	+	-
Isoleucine	3.72 (t)	-	+
Acetone	2.21 (s)	+	-
Pyruvate	2.39 (s)	+	-
Succinic acid	2.63 (s)	+	-
Malic acid	2.76 (dd, J = 13.5, 2.5 Hz	+	-
Aspartate	2.82 (dd, J = 17.0 Hz, 8.5 Hz)	+	-
Tyrosine	3.02 (dd)	+	-
O- Phosphoetanolamine	3.18 (m)	-	+
Oleanolic acid	3.21 (dd, J = 4.0, 3.5 Hz)	-	+
Choline	δ 3.27 (s)	-	+
Tryptophan	3.31 (dd, J= 13.4, 5.0 Hz)	+	-
3-Hydroxyphenylacetate	3.5 (s)	-	+
1.3-Dihydroxyacetone	3.58 (d, J = 4.2 Hz)	-	+
Fructose	3.78–3.82 (m)	-	+
Glutamate	3.75(m)	-	+
Uridine	3.92 (dd, J = 12.0, 3.8 Hz)	+	-
Glucose	5.20 (d, J = 3.5 Hz)	-	+
Sucrose	5.42 (d, J = 3.5 Hz)	-	+
Uracil	5.78(d, J = 7.6 Hz)	+	-
Cytosine	5.92(d, J = 8.0 Hz)	+	-

J; NMR J-coupling or nuclear spin-spin coupling; m; multiplet, t; triplet, d; doublet, dd; doublet of doublet; singlet. -; that particular compound is not present, +; the compound is present.

## Data Availability

The data used to support the findings of this study are included within the article. Any other data can be available upon request.

## Supplementary Materials

The following supporting information can be downloaded at: https://www.mdpi.com/article/10.3390/foods12101971/s1, Figure S1: LAB from fermented and pasteurized coconut milk appearance in MRS agar; Figure S2: yeast and molds from fermented coconut milk appearance in DRBC agar; Figure S3: Coliform and *E. coli* from coconut milk appearance in coliform agar; Figure S4: The representative ^1^H NMR spectra of fresh and fermented coconut milk; Figure S5: The regression coefficient of the correlation showing PLS model validation.
