# Supplementary material for: Characterization of Physicochemical, Biological, and Chemical Changes Associated with Coconut Milk Fermentation and Correlation Revealed by 1H NMR-Based Metabolomics
Source: Foods. 2023 May 12;12(10):1971. doi: 10.3390/foods12101971 (PMC10217123; doi:10.3390/foods12101971)
Supplement: Supplementary file 1 [file foods-12-01971-s001.zip › foods-1893965-supplementary.pdf]

Supplementary Materials

Article

Chemical and biological changes of fermented coconut milk revealed by ^{1}H NMR-based metabolomics

Wasim S.M Qadi[1], Ahmed Mediani[2,*], Khaled Benchoula[3], Wong Eng Hwa[3], Norazlan Mohmad Misnan[4], Norrakiah Abdullah Sani[1,*]

[1] Department of Food science, Faculty of Science and Technology, Universiti Kebangsaan Malaysia 43650, Bangi Selangor, Malaysia
[2] Institute of Systems Biology (INBIOSIS), Universiti Kebangsaan Malaysia, 43600, UKM Bangi, Selangor, Malaysia
[3] School of Medicine, Faculty of Health and Medical Sciences, Taylor's University, 1, Jalan Taylors, 47500 Subang Jaya, Selangor, Malaysia;
[4] Herbal Medicine Research Centre, Institute for Medical Research, National Institutes of Health, 40170 Shah Alam, Selangor, Malaysia

* Correspondence: norrasani@ukm.edu.my; ahmed@ukm.edu.my; Tel.: +603-8921 4546

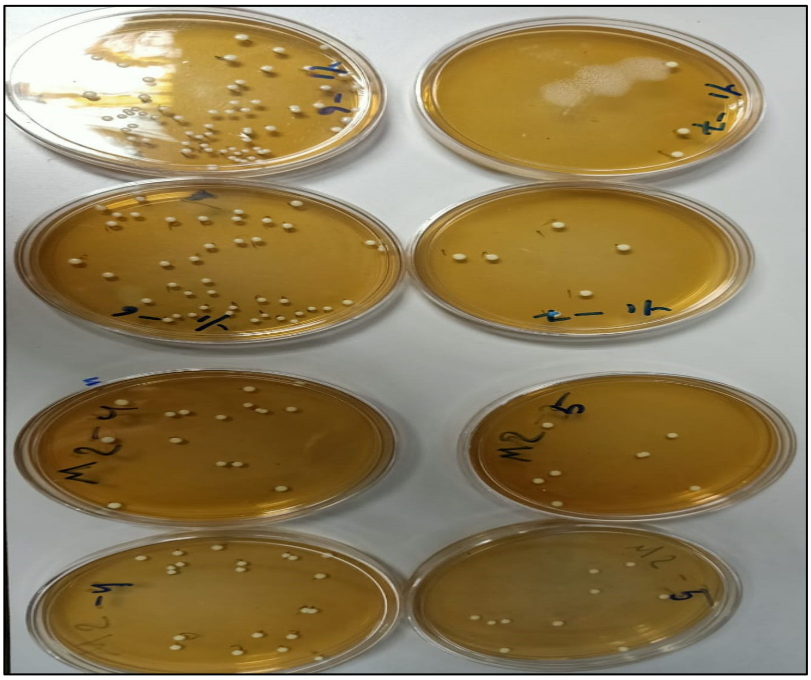

Figure S1. LAB from fermented and pasteurized coconut milks appearance in MRS agar.

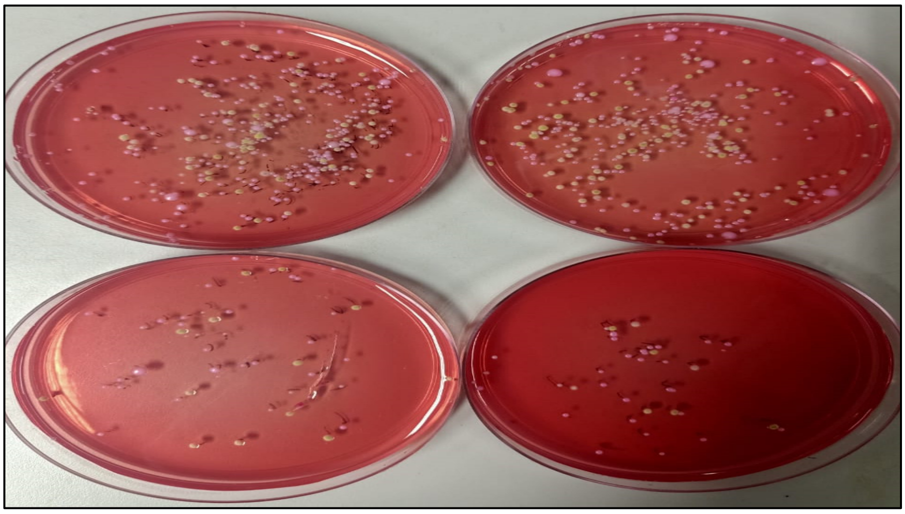

Figure S2. yeast and molds from fermented coconut milk appearance in DRBC agar.

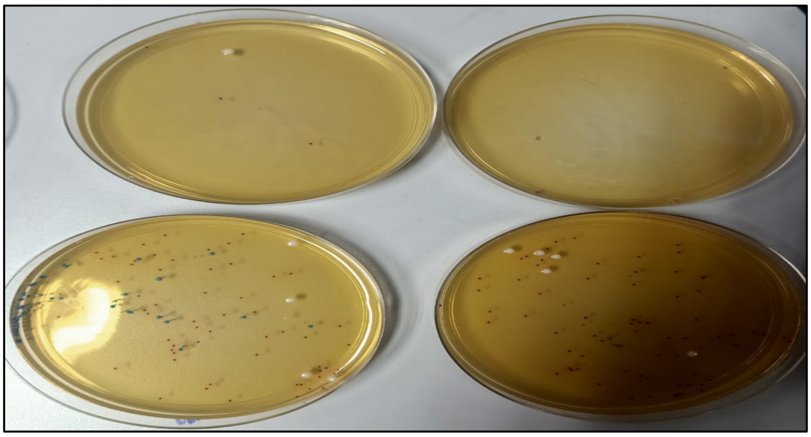

Figure S3. Coliform and *E. coli* from coconut milk appearance in coliform agar.

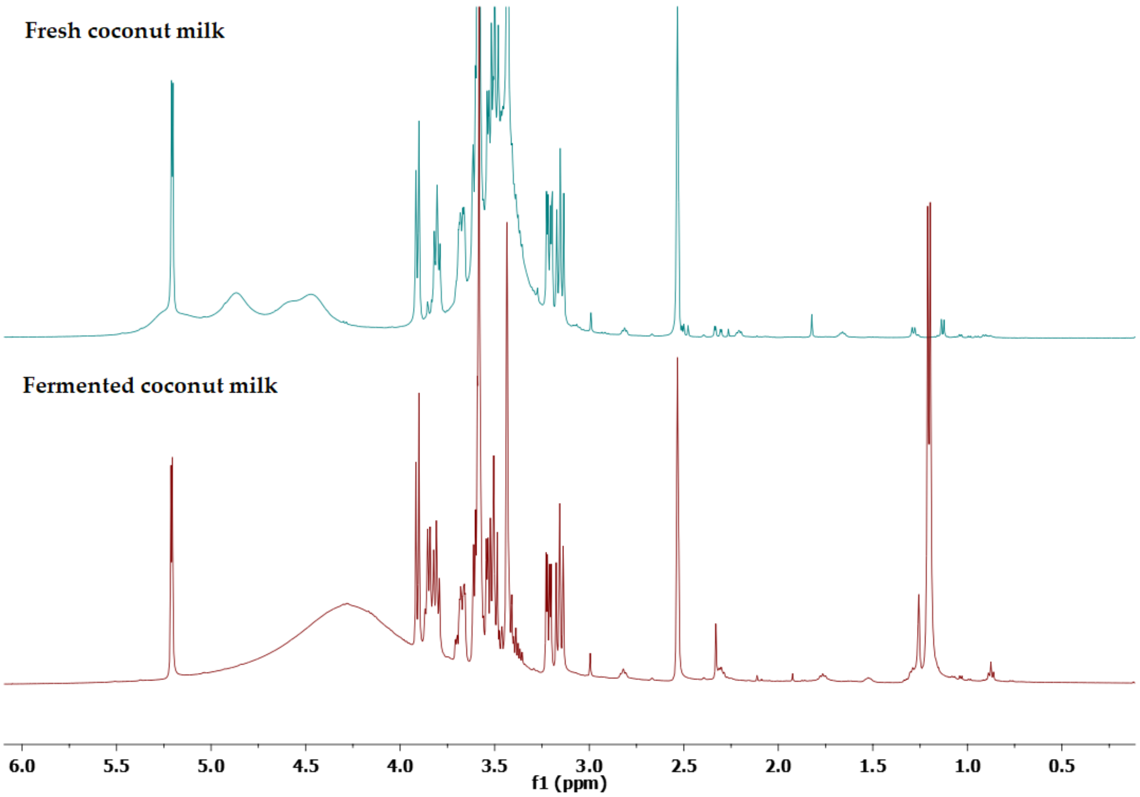

Figure S4. The representative ^{1}H NMR spectra of fresh and fermented coconut milk.

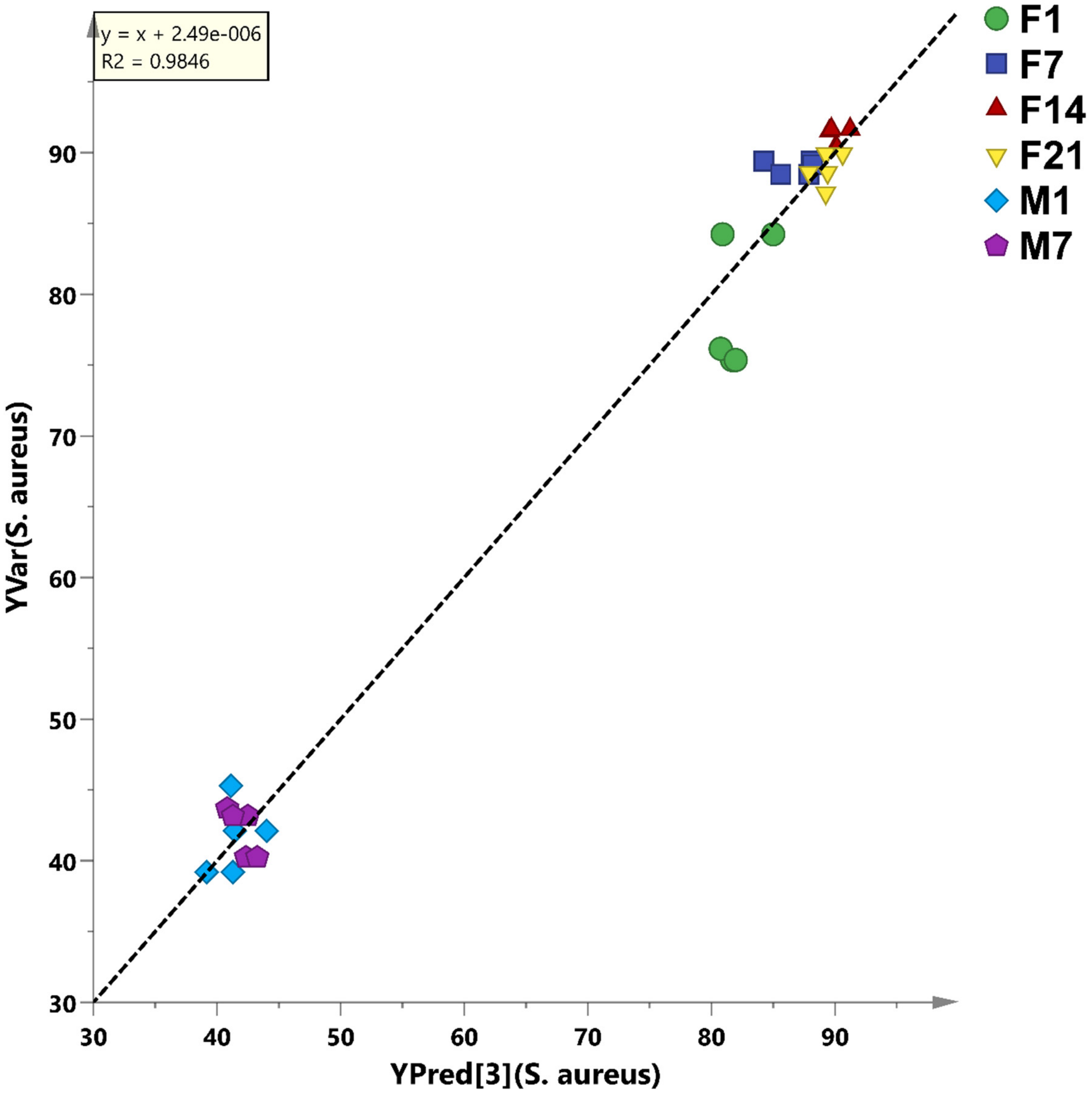

Figure S5. The regression coefficient of the correlation showing PLS model validation
